# A 3D printed cast for minimally invasive transfer of distal radius osteotomy: a cadaver study

**DOI:** 10.1007/s11548-021-02310-7

**Published:** 2021-01-21

**Authors:** G. Caiti, J. G. G. Dobbe, S. D. Strackee, M. H. M. van Doesburg, G. J. Strijkers, G. J. Streekstra

**Affiliations:** 1 Department of Biomedical Engineering and Physics, Amsterdam Movement Sciences, Amsterdam UMC, University of Amsterdam, Amsterdam, The Netherlands; 2 Department of Plastic, Reconstructive and Hand Surgery, Amsterdam Movement Sciences, Amsterdam UMC, University of Amsterdam, Amsterdam, The Netherlands

**Keywords:** Radius osteotomy, Surgical guide, 3D printing, Minimally invasive navigation, Computer-assisted orthopedic surgery

## Abstract

**Purpose:**

In corrective osteotomy of the distal radius, patient-specific 3D printed surgical guides or optical navigation systems are often used to navigate the surgical saw. The purpose of this cadaver study is to present and evaluate a novel cast-based guiding system to transfer the virtually planned corrective osteotomy of the distal radius.

**Methods:**

We developed a cast-based guiding system composed of a cast featuring two drilling slots as well as an external cutting guide that was used to orient the surgical saw for osteotomy in the preoperatively planned position. The device was tested on five cadaver specimens with different body fat percentages. A repositioning experiment was performed to assess the precision of replacing an arm in the cast. Accuracy and precision of drilling and cutting using the proposed cast-based guiding system were evaluated using the same five cadaver arms. CT imaging was used to quantify the positioning errors in 3D.

**Results:**

For normal-weight cadavers, the resulting total translation and rotation repositioning errors were ± 2 mm and ± 2°. Across the five performed surgeries, the median accuracy and Inter Quartile Ranges (IQR) of pre-operatively planned drilling trajectories were 4.3° (IQR = 2.4°) and 3.1 mm (IQR = 4.9 mm). Median rotational and translational errors in transferring the pre-operatively planned osteotomy plane were and 3.9° (IQR = 4.5°) and 2.6 mm (IQR = 4.2 mm), respectively.

**Conclusion:**

For normal weight arm specimens, navigation of corrective osteotomy via a cast-based guide resulted in transfer errors comparable to those using invasive surgical guides. The promising positioning capabilities justify further investigating whether the method could ultimately be used in a clinical setting, which could especially be of interest when used with less invasive osteosynthesis material.

## Introduction

Distal radius malunion is a complication frequently arising after a conservatively treated fracture of the distal radius [1]. When clinically relevant, the malunion is surgically treated by corrective osteotomy (CO) surgery [2]. The purpose of CO is to reconstruct as best as possible the original bone alignment by cutting the bone at the level of the old fracture (osteotomy) and then repositioning and fixating the bone segments to a near-anatomical alignment. State-of-the-art CO techniques combine the utilization of three-dimensional (3D) pre-operative virtual planning and 3D printed patient-specific instruments (PSI’s). With virtual pre-operative planning, 3D virtual models of the affected and the contralateral healthy radius anatomy are generated from a bilateral computed tomography (CT) scan of a patient’s forearm [3]. Furthermore, starting from these 3D virtual models, it is possible to design PSI’s exactly fitting the bone anatomy and featuring a cutting slit and drilling holes to orient the surgical tools as preoperatively planned [4–7]. Currently, the use of PSI’s is growing in popularity because they offer a straightforward method for surgical navigation [8], they are accurate in transferring the virtual plan to the patient [9], and are becoming more affordable thanks to advances in design software and developments in additive manufacturing technology [10].

Recently, a minimally invasive patient-specific rimmed wedge implant has been proposed for repositioning and fixation in corrective osteotomy of the distal radius, which exactly fits the osteotomized bone segments in the planned position [11]. Although far from being used in a clinical setting, this procedure requires accurate osteotomy, since the wedge fits in between the bone segments at a single planned location. The osteotomy in this approach could be performed using PSI’s. However, a drawback of PSI’s is that complete dissection of the soft tissues covering the bone surface is generally required in order to ensure an accurate fit of the PSI on the target bone. This is undesired because with complete soft tissue dissection the patient is expected to experience slower recovery, inferior functional results and a poorer forearm appearance after surgery [12, 13] (Level of evidence IV). A navigation technique that positions and orients the surgical saw without considerable soft tissue dissection would better fit the minimally invasive approach of corrective osteotomy using a rimmed wedge implant.

One of the possible approaches to minimize the invasiveness of the osteotomy procedure and to eliminate the need for a large incision is to use an external cutting guide to be mounted on percutaneously inserted pins. Percutaneous pinning is a common procedure in hand surgery and is frequently performed in order to fixate carpal or distal radius fractures [14]. Systems for minimal invasive navigation of percutaneous pinning of carpal bones have been previously proposed [15–17]. In systems targeting percutaneous pinning of the scaphoid, the wrist is set in a fixed position through a stabilization platform or stereotactic frame which features a local coordinate reference (LCR). Then, image-guided surgical navigation can be performed by tracking the LCR on the wrist stabilization frame during image-guided surgery [15–17]. These systems permit minimally invasive navigation because markers are not directly attached to the bone but to the wrist-stabilization frame. However, the positioning accuracy of these stabilization platforms is often unknown [16, 17] or suffers from large patient-device motion caused by drilling (up to on average 16 mm and 6.5°) [15].

The aim of this cadaver study is to propose and evaluate a minimally invasive method for percutaneous pinning that serves to place a cutting guide for navigation of the surgical saw for corrective surgery of the distal radius. The proposed method consists of a 3D-printed cast that enables percutaneous pin insertion and positioning of an external cutting guide that is slid over the pins after cast removal. We evaluate the repeatability of repositioning the radius in the cast and its dependency on the amount of soft tissue surrounding the bone. We further evaluate the accuracy and precision of transferring the virtual pins and the osteotomy cut using the proposed surgical system.

## Materials and methods

**Fig. 1:**
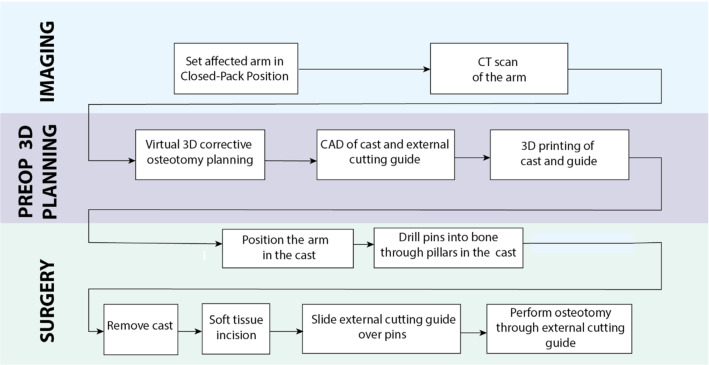
Pre and intra-operative workflow for cast-guided osteotomy

The proposed method is based on the following workflow (Fig. 1): After positioning the arm in a reproducible position (detailed in section 2a), a preoperative bilateral CT scan is acquired. Then, the 3D pre-operative planning is performed as described in [18–22], where the osteotomy plane and bone repositioning are defined. Next, the cast and the external cutting guide are designed, via Computer Aided Design (CAD) software, and 3D printed. In this study, image analysis and 3D printing took approximately 24 h. During surgery, the cast is applied. Percutaneous pinning of the radius is performed through drilling pillars integrated on the cast surface. After cast removal and preliminary incision of the skin and soft tissues, the external cutting guide is placed over the pins and the osteotomy can be performed through a slit in the guide.

**Fig. 2:**
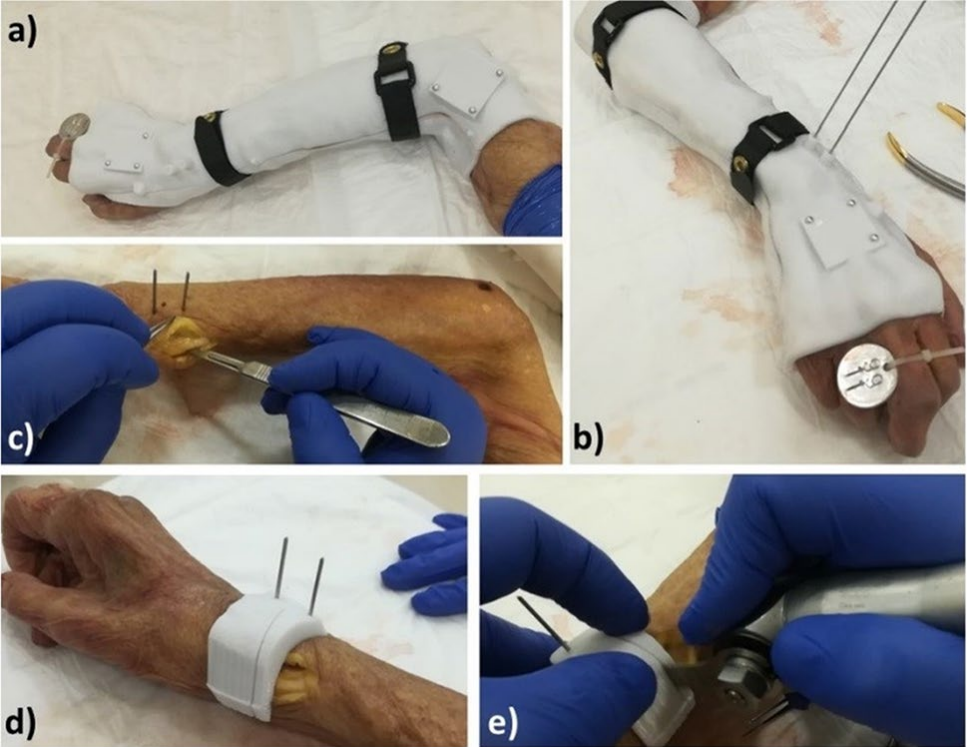
Practical steps during the surgical procedure. **a** The arm is inserted in the cast; **b** two self-drilling/self-tapping pins are percutaneously drilled through the drilling slots; **c** after cast removal, a small incision is performed; **d** The external cutting guide is slid over the previously fixed pins; **e** osteotomy is performed thorough the slit in the cutting guide

Figure 2 shows steps in the surgical procedure.

In the following subsections, the rationale behind the cast design (A), the experiments to investigate the reproducibility of positioning the arm in the cast (B), and the method to quantify the surgical transfer accuracy (C) are presented.

### Cast and guide design

**Fig. 3:**
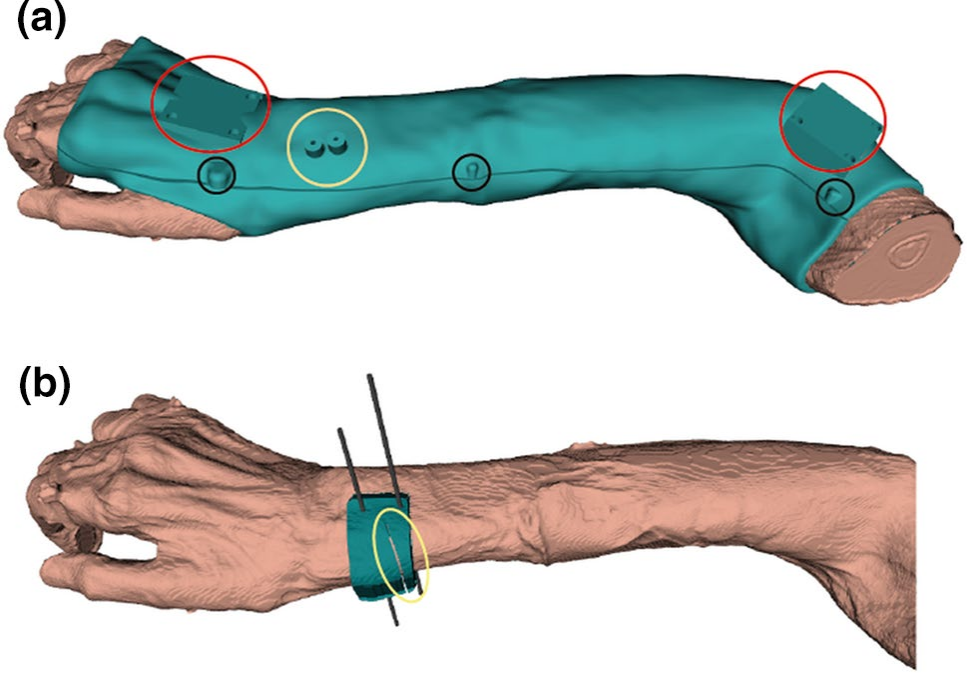
**a** The arm is placed in a stable and reproducible position (ulnar deviation ~ 35°, fingers flexed and elbow at ~ 45**°** flexion). The cast features distal and proximal marker tools (red circle); two drilling pillars (yellow circle) and snap-lock pins (black circles); **b** after inserting self-drilling/self-tapping pins into the bone through the pillars in the cast, and after cast removal, an external cutting guide is slid over the pins. The guide features a slit (marked in yellow) to guide the surgical cutting blade

In this study, five cadaver arm specimens were included. Each cast-based surgical guide was designed starting from a CT image of a fresh-frozen cadaver arm after thawing for 24 h. One of the main challenges of the presented method was to reproduce the preoperatively scanned position of the radius relative to the cast during surgery. Therefore, a stable and reproducible forearm position was chosen when imaging the arms. A high reproducibility of positioning was expected in the so-called Closed-Pack Position (CPP) in which the contact surface between articulating bones is maximal resulting in the highest mechanical stability. For the wrist joint a CPP is obtained with the wrist in maximal (approx. 35˚) ulnar deviation. In order to avoid shifting of the cast along the radial axis, we further chose to flex the fingers and the elbow at approx. 45° (Fig. 3a). After arm replacement (10 ×), a scan was acquired with either a Brilliance 64-channel scanner (Philips Healthcare, Best, The Netherlands) or with a Sensation 64-channel scanner (Siemens, Munich, Germany). Comparable dedicated scanning protocols were adopted for the two scanners (tube voltage 120 kVp; exposure: 500 mAs; slice thickness 0.90 mm) and (Tube Voltage 120 kVp; exposure: 400 mAs; slice thickness 0.75 mm) with isotropic voxel spacing of 0.45 mm, respectively. From each acquired CT scan, the radius bone and the entire arm were segmented using custom software, as described in [23]. Each cast was designed via Autodesk (Autodesk Inc., San Rafael, California, USA) and was composed of two matching halves. In order to fit the two halves together intraoperatively, 6 snap-lock aluminum pins (3 on the ulnar and 3 on the radial side of the cast) were placed on the cast edge (Fig. 3a). Two marker tools were included in each cast, representing a distal and proximal Local Coordinate System (LCR). Each marker tool contained three aluminum spheres (5 mm diameter) in an orthogonal configuration. These local coordinate systems served to quantify deformation of the cast and positioning of the radius bone with respect to the cast when repetitively placing the arm in the cast (detailed in section B). Each cast featured two pillars to guide percutaneous pin insertion targeting the dorsal aspect of the distal radius. To this end, self-drilling/self-tapping pins (Stryker, Michigan, United States, diameter 3 mm, total length 80 mm, thread length 20 mm) were used. Drilling pillars were placed parallel to each other and perpendicular to the normal of the chosen cutting plane (Fig. 3a). This made the osteotomy cut irrespective of guide placement along the pins. Care was taken to make cast removal feasible for each of the applicable drill paths. For osteotomy guidance, external cutting guides were designed (Fig. 3b) using the same custom software as described above. Both casts and external guides were 3D printed in Polycarbonate-ISO (PC-ISO, certified ISO 10,993, United State Pharmacopeia -USP- Class VI), with a Stratasys Fortus 450 mc Fused Deposition Modelling (FDM) printer (Stratasys, Eden Prairie, Minnesota, USA). Printer accuracy was ± 0.127 mm in all directions. The thickness of the cast was set to 4 mm. The material PC-ISO is sterilizable using gamma radiation or ethylene oxide gas [24].

### Reproducibility of arm replacement

**Fig. 4:**
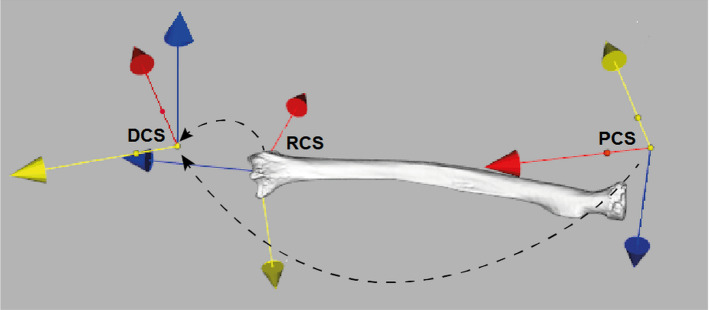
For each CT image, after segmentation, three Coordinate Systems (CS’s) are defined: an anatomical CS for the radius bone (RCS) and CS’s for the distal and for the proximal marker tools (DCS and PCS) on the cast. The transformation from RCS-to-DCS provides the relative position of the radius with respect to the cast. The transformation from PCS-to-DCS represents the cast deformation

Each cadaver specimen was placed and removed 10 times in the 3D printed cast to quantify the reproducibility of radius positioning relative to the cast. After each arm placement, a CT scan was acquired. The 3D polygonal model of the radius bone (see section A) was registered to each CT scan [23]. In agreement with previously reported study [9], a radius right-handed coordinate system (RCS) was defined for each segmented radius (see section A). In brief, the *z*-axis coincided with the principal axis of inertia of the radius model, the x-axis was directed toward the styloid process and the y-axis was consequently oriented to establish an orthogonal triplet of vectors (Fig. 4). Markers tools on the surface of the cast were used to provide local CS’s for the cast. In brief, the metal spheres included in the marker tools were segmented by automatic thresholding which enabled automatic extraction of the orthogonal axes that define the local coordinate systems for the two marker tools, since the distance between the three spheres in each marker tool was unique, as described in [19]. In this way a proximal coordinate system (PCS) and a distal coordinate system (DCS) were defined on the cast (Fig. 4). The DCS served as reference coordinate system for expressing:

1. The relative displacement of the radius into the cast, represented by variability in the RCS-to-DCS transformation
2. The deformation of the cast during the experiment, represented by changes in the PCS-to-DCS transformation.

Each transformation matrix enabled calculations of the repositioning parameters, in six degrees of freedom (DOF) (3 rotations and 3 translations). Repetitive arm placements provided the reproducibility in the repositioning parameters and cast deformation. Errors are reported as the difference from the mean parameter.

The total translation (Δ*T*) and the total rotation (Δ*R*) errors, respectively, defined as the Euclidian norm of the translation and rotation parameters [25], were also calculated. We hypothesized that positioning of the radius in the cast and the subsequent drilling and cutting accuracy are dependent on the amount of rigid bony protrusions providing stability to the cast. The reproducibility may therefore deteriorate if more soft tissue covers these bony protrusions. In order to confirm our hypothesis, we report the positioning errors as a function of the soft-tissue percentage (${ST}_{\%}$) of the arm specimen, which was calculated as follows:

$${ST}_{\%}=\frac{{V}_{\mathrm{arm}}-{V}_{\mathrm{bone}}}{{V}_{\mathrm{arm}}}\cdot 100\%$$

where ${V}_{\mathrm{arm}}$ is the total volume of the segmented arm and ${V}_{\mathrm{bone}}$ is the volume of all the segmented skeletal structures in the specimen. Mid-upper arm circumference was also used as an estimate of the body mass index (BMI) of the subjects [26]. Selection of the arm specimens was therefore based on the soft tissue content of the arms to obtain a range of soft-tissue percentages among the set of specimens.

### Quantifying cutting and drilling error

**Fig. 5:**
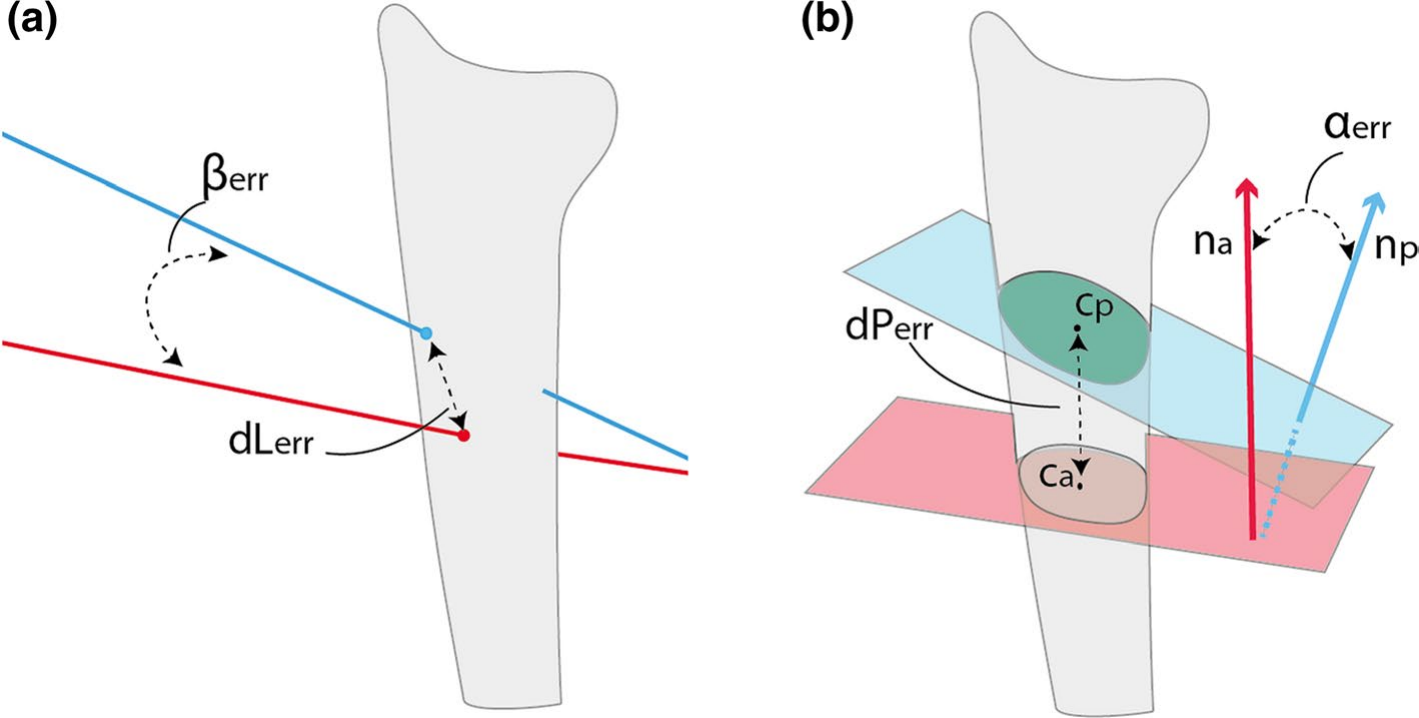
**a** The planned (blue) and the achieved (red) drilling lines. The displacement error (dL_err_) is defined as the distance between the entry point of each line into the bone polygon, the angular error (*α*_err_) is defined as the angle between the two line versors; **b** the planned (blue) and the achieved (red) cutting planes and the corresponding normal vectors (*n*_a_, *n*_p_). The angular error (*β*_err_) is defined as the angle between *n*_a_ and *n*_p._ The distance between the centroids (*c*_a_, *c*_p_) of the achieved and the planned bone cross sections represents the displacement error (dP_err_)

For each of the specimens, the overall procedure consisting of drilling through the cast and then performing the osteotomy through the external cutting guide was carried out by an experienced surgeon. After removal of the pins, a final CT scan of the drilled and cut bones was acquired. The images were analyzed in the following way: the distal and proximal bone segments were segmented and registered to the preoperative image that was used for planning. This enabled comparisons of the achieved drill-hole positions with the planned positions, as well as the achieved osteotomy cut with the planned cut. To this end, a distal and a proximal line was manually fit through the respective screw entry and exit points on the bone in 3D space. The difference between the planned and achieved pin placement was calculated as the distance (dL_err_) between the planned and achieved entry point of the drilling lines into the bone and as the angular offset (*α*_err_) between the line directions (Fig. 5a). To quantify the osteotomy error, the achieved osteotomy plane was identified by manually placing three points on the surface of the proximal bone segment and then fitting a plane through these points. The difference between the planned and actual cutting plane was calculated as the distance (dP_err_) between the centroids of the planned and the achieved bone cross sections [27] and as the angular error (*β*_err_) between the normals of the planned and the achieved cutting plane (Fig. 5b).

## Results

### Reproducibility of arm positioning

**Fig. 6:**
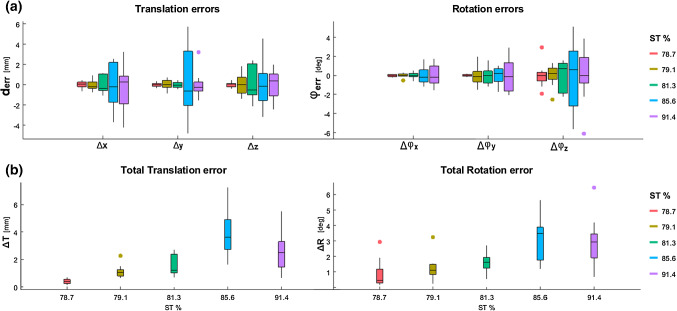
Boxplots representing the summary statistics (median and IQR) of the 6 DoF repositioning errors in the 5 patient-specific casts, using specimens with increasing percentage of soft tissue (ST). On each box, the central mark indicates the median; the whiskers represent the 25th (Q1) and 75th (Q3) percentiles range of each parameter. The dots are data considered outliers (< Q1 − 1.5*IQR or > Q3 + 1.5*IQR).: **a** The 6 DOF translational rotational errors; **b** The total translation and rotation errors

Quantification of the arm and bone volume from the CT scans of the five cadaver specimens, provided a soft tissue (ST) content of, respectively, 78.7%, 79.1%, 81.3%, 85.6% and 91.4%. The second and the third arms (in order of ST content) originated from the same individual. Mid-upper arm circumference values were correlated with BMIs of 14, 23, 23, 28 and 31 which roughly categorize the subjects as, respectively, falling in the underweight, normal (2 ×), overweight and obese ranges, according to [28]. For equivalent judgment of the 6 DoF parameters all left radii were mirrored. Boxplots reporting the median and the Interquartile Range (IQR) of the three translation parameters (${\Delta }_{x}, {\Delta }_{y}, {\Delta }_{z}$) and of the three rotation parameters (${\Delta }_{{\varphi }_{x}}, {\Delta }_{{\varphi }_{y}}, {\Delta }_{{\varphi }_{z}}$) are reported in Fig. 6a, b, respectively. The total rotation and translation errors obtained with the casts in the five cases are shown in Fig. 6c, d. While positioning errors appear to remain in the range ± 2 mm/deg for specimens composed up to 81.3% by ST, we can see an increase in the positioning errors for the specimens categorized as overweight and obese (85.6 and 91.4% ST).

**Fig. 7:**
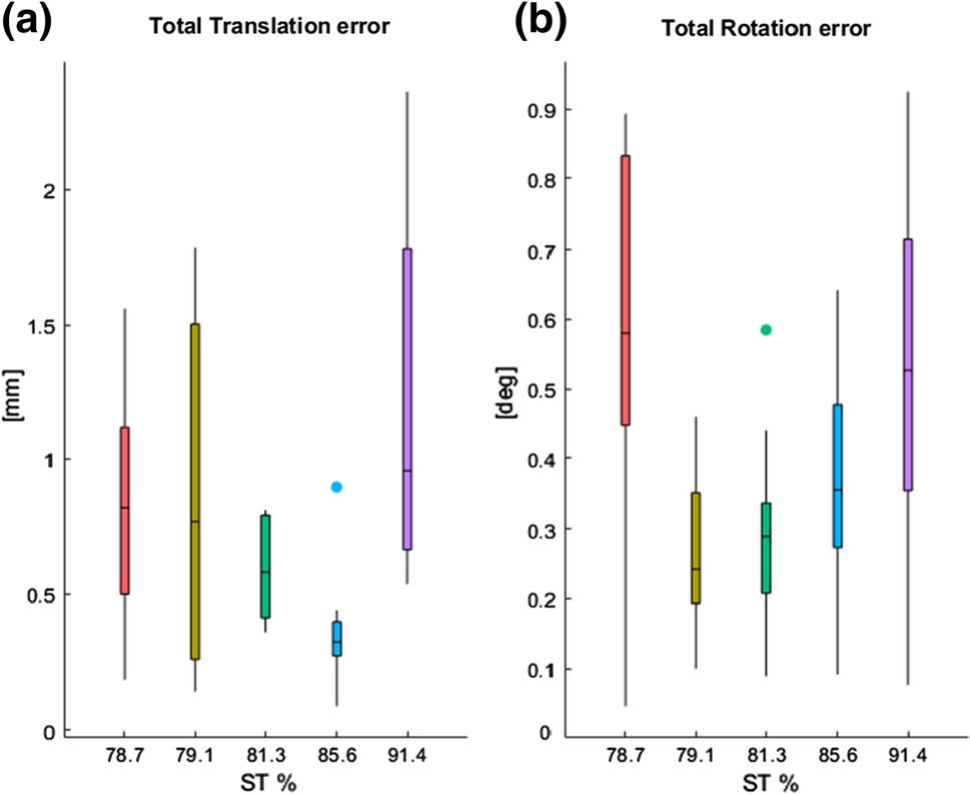
Cast deformation represented by box-plot showing a) the total translation error ($\Delta T$) and total rotation error ($\Delta R$) occurring between the two local marker tools on the cast

Variability of arm repositioning was partially caused by elastic deformation of the cast devices. Figure 7 shows summary statistics of the total translations and total rotations errors occurring between the marker tool coordinate systems (DCS and PCS) on the five casts during the repositioning experiment. Deformation of the cast contributed up to 2 mm to the overall repositioning translation error and up 1 degree to the total rotation error. No trend could be observed between deformation errors and specimens’ soft tissue content (ST%).

### Transfer error of drilling and cutting

**Fig. 8:**
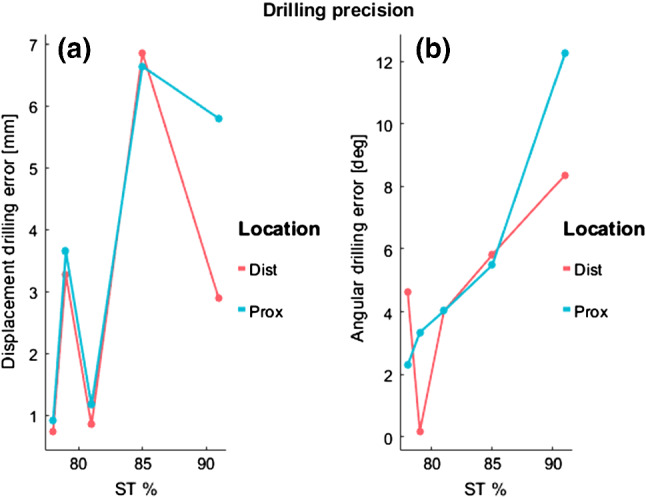
Error parameters in transferring the drill holes to the specimens as a function of soft tissue percentage (ST%). **a** Displacement error of the achieved drilling lines (proximal and distal) from the planned position; **b** Angular error between the planned and the achieved drilling lines

The five cast-guided osteotomies were performed as described in section 2a. In one case (91.4% ST specimen), the drilling pillars were too tight for the chosen pins. Therefore, in the attempt to enlarge them, the pillars were damaged by the surgical drill. No plastic debris of the guide was observed at the surgical site in any of the cases. The errors in achieving the planned drilling trajectories across the five cases are depicted in Fig. 8. Displacement errors were similar for distal and proximal drilling lines in the same subject. An exception can be seen in the case of the specimen with 91.5% ST, which could be explained by the damage caused to the drilling pillars of the cast. For cases up to 81.3% ST, displacement of drilling lines from the planned position did not exceed 4 mm. Angular drilling errors were below 6° for all the cases where the drilling guide remained intact.

**Fig. 9:**
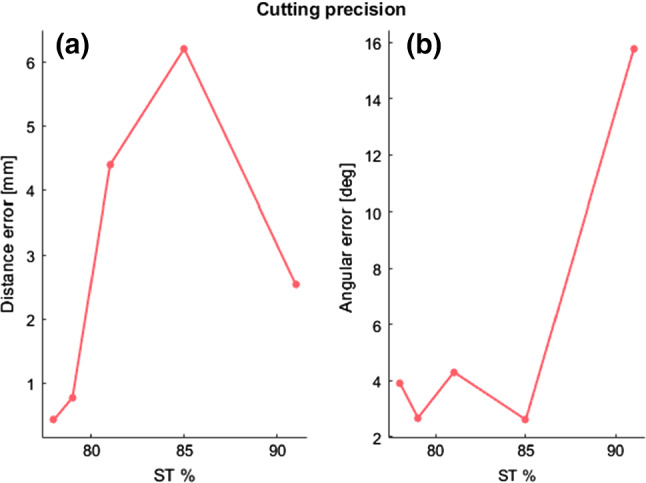
Error in transferring the osteotomy across the five specimens with increasing percentage of soft tissue (ST), **a** distance error between the centroids of the bone cross-sections achieved with the planned and the achieved cutting planes; **b** angular error between the normal vector of the planned and *the achieved osteotomy planes*

The error in transferring the osteotomy through the custom cutting block in the selected cases is described in Fig. 9. Displacements errors in performing the planned osteotomy (Fig. 9a) were up to 4.5 mm for cases up to 81.3% ST. Angular errors (Fig. 9b) remained below 4.2° in all the cases except for the 91.5% ST.

## Discussion

The aim of the present cadaver study was to propose and evaluate a novel minimally invasive approach to quickly and easily navigate a corrective osteotomy of the distal radius. We designed an innovative 2-step surgical system composed of an externally fitting cast to guide dorsal insertion of two percutaneous self-drilling/self-tapping pins in the distal radius, and an external cutting guide to be slid over the pins. We presented the device together with the step-by-step surgical procedure which was performed on five cadaver specimens.

The error of repositioning the radius within the cast was evaluated in 6 Degrees of Freedom with a CT-based technique. In specimens with a normal body weight, the repositioning error was in the range ± 2 mm and 2°. This is comparable to the errors obtained when manually positioning invasive guides on the volar radial surface as quantified in our previous study with the same image-based technique [9]. Except for one case (95.1 ST %) where a surgical error damaged the drilling guide on the cast, the technique performed particularly well in achieving the planned osteotomy with errors limited to 4.4 mm and 4.2°. Errors in achieving the correct osteotomy orientation were smaller than the angular drilling errors. This reduced error could be explained by the use of the cutting block, which probably corrected the pins parallelism while performing the bone cut. In normal weight subjects, the proposed device has therefore the potential to render corrective osteotomy surgery less invasive, especially if used in combination with minimally invasive osteosynthesis material such as a custom wedge implant [11]. This patient-specific titanium wedge insert features two rims to stabilize the bone segments, and two drilling holes for screw fixation. The implant is designed to align the bone segments as preoperatively planned. Further study is needed to demonstrate the overall feasibility and accuracy of such an approach.

In this cadaver study, we mainly focused on the error of performing an osteotomy using a minimally invasive 3D printed cast. The positioning results seem promising, which justifies further investigating navigated osteotomy surgery using the cast-based guiding system in future research. However, the method is not ready for clinical use. There are several limitations that require further investigation. In our study, we relate positioning reliability to soft tissue volume while skin motion may have an independent influence that is worthwhile investigating. Although the proposed method for bone repositioning seems promising, it performed clearly better in specimens with a lower fat content. Deformation of the cast itself (up to 2 mm, 1°) contributed to the total error that we observed. Investigating alternative cast materials with better mechanical properties and using different designs of the cutting block may contribute to improved accuracy of the method. In this study we used a limited number of cadaver specimens. This hampers drawing a final conclusion on the clinical value of the method. For future research we therefore recommend optimizing cast design, avoiding obstruction of the surgical target giving a better surgical approach that avoids tendon or nerve injury, and evaluation of these techniques using more cadaver specimens. We also recommend investigating advantages, disadvantages, and potential risks and complications related to using the proposed cast-based navigation technique, and to compare these with established navigations techniques, including invasive guides and optical navigation. If the method is ultimately suitable for clinical evaluation, we recommend investigating whether the minimally invasive approach is substantially better from the patient’s perspective compared to using invasive techniques.

The main challenge of this study was related to the error of repositioning the radius in the custom cast, which was designed to fit a deformable arm. Similar attempts of fitting external surgical guiding systems to the patient body have been reported in the literature. However, despite a favorable clinical outcome, the positioning accuracy of the devices has rarely been reported. For example, Li et al*.* used 3D printed PSI’s to guide percutaneous vertebroplasty with a customized template that was fitting the back of the patient and featured drilling sleeves to guide the needle tip [29]. The customized template was used in combination with C-arm fluoroscopy in one patient case with good surgical outcome, but no methodological drilling errors were reported. Yin et al*.* reported the use of a 3D printed short cast extending to the wrist of the patient to guide percutaneous pinning for scaphoid fracture fixation [30]. Although design details and the surgical procedure were extensively described, neither postoperative evaluation nor the fixation error were quantified. Our study is therefore the first quantitative evaluation of the feasibility of using an external guiding device. Furthermore, compared to the previously reported studies our method does not expose subjects to intraoperative radiation.

## Conclusions

We developed a minimally invasive device for the transfer of a 3D planned corrective osteotomy of the distal radius and evaluated the method experimentally. For cadaver specimens with a low and normal soft tissue content, precision in positioning the arm in the cast was comparable with the positioning accuracy of invasive PSI’s for the distal radius. The promising positioning capabilities justify further investigating whether the method could ultimately be used in a clinical setting.
